# *GsZIP7*, a Zinc/Iron-Regulated Transporter Protein from Wild Soybean, Confers Enhanced Sensitivity to Alkaline Stress

**DOI:** 10.3390/plants15142152

**Published:** 2026-07-13

**Authors:** Zichun Wei, Chengbo Zhang, Yangyang Fang, Xiaoxia Jin, Jia Cui, Chao Chen

**Affiliations:** Department of Chemistry and Molecular Biology, School of Life Science and Technology, Harbin Normal University, Harbin 150025, China

**Keywords:** zinc/iron-regulated transporter-like protein, wild soybean, alkaline stress, *GsZIP7*

## Abstract

Zinc/iron-regulated transporter proteins (ZIPs) are involved in the transport of zinc and iron, maintenance of ion homeostasis, and regulation of plant responses to abiotic stresses. Although previous studies have identified members of the *ZIP* gene family and screened for alkaline-responsive *ZIP* genes in wild soybean (*Glycine soja*), the functional implications of these genes under alkaline stress conditions remain to be fully elucidated. The present study aimed to identify the *GsZIP7* gene and investigate its regulatory role in *Arabidopsis* and wild soybean under alkaline stress. The results showed that *GsZIP7* was highly expressed in roots and young stems. *GsZIP7* exhibited differential expression in response to alkaline stress, zinc deficiency, or iron deficiency in wild soybean. In addition, heterologous expression of *GsZIP7* in yeast mutants increased tolerance to iron- and zinc-deficient conditions. Overexpression of *GsZIP7* in *Arabidopsis* resulted in reduced root growth, decreased fresh weight, and a weakened antioxidant defense system. Furthermore, *GsZIP7*-overexpressing soybean hairy roots showed increased sensitivity to alkaline stress, whereas *GsZIP7*-RNAi lines exhibited enhanced tolerance. Together, these findings indicate that GsZIP7 negatively regulates alkaline tolerance in plants.

## 1. Introduction

As a major abiotic constraint, alkaline stress impairs plant growth and development [1]. Alkaline stress impairs plant growth through multiple mechanisms: elevated Na^+^, high pH, and HCO_3_^−^, which disrupt cellular metabolic balance, compromise cytosolic pH homeostasis, alter ion homeostasis, and impair oxidative stress responses [2,3]. Particularly, alkaline stress triggers Na^+^ influx and disrupts Na^+^/K^+^ homeostasis, while high pH precipitates soil essential cations (Ca^2+^, Mg^2+^, Fe^2+^, Zn^2+^ and Cu^2+^) from the soil solution [3]. This process impairs the root nutrient reuse capacity and nutrient absorption efficiency, ultimately leading to plant nutrient deficiency [4]. To counteract these effects, plants activate ion transport systems to maintain pH homeostasis and enhance antioxidant defenses to mitigate oxidative stress [5].

The *ZIP* family of zinc and iron transporters is essential for plant growth and development by regulating cellular Zn^2+^ and Fe^2+^ homeostasis [6]. Their expression directly controls micronutrient uptake and utilization, thereby determining plant growth performance [7]. ZIP proteins transport Zn^2+^, Fe^2+^, and other divalent cations (such as Cd^2+^ and Cu^2+^) using a proton gradient, enabling root acquisition and intracellular ion distribution [8]. Structurally, they comprise 300–500 amino acids and feature eight transmembrane domains (TMs). A hydrophilic loop between TM3 and TM4 functions as a metal-binding site [9]. Both the N- and C-termini face the cytoplasm, which is a unique topological feature of the ZIP transporter family [10].

The *ZIP* family mediates the active transport of Zn^2+^, Fe^2+^, and other divalent cations [11]. Zn^2+^ and Fe^2+^ can coordinate with amino acid residues including histidine, aspartic acid, and glutamic acid at the conserved metal-binding sites within ZIP transporters. The metal-bound protein undergoes a conformational transition across the lipid bilayer, thereby mediating directional ion translocation [12]. The *ZIP* family typically does not function independently, but operates within a metal homeostasis regulatory network. During iron uptake, the FIT/bHLH transcription factor activates AHA2 to enhance rhizosphere acidification, and reduces Fe^3+^ to Fe^2+^, which subsequently enables ZIP transporters to mediate the influx of Fe^2+^ into cells [13,14]. *AtIRT1* was the first identified iron transporter in *Arabidopsis*, where it mediates root iron uptake [15]. In rice, OsZIP proteins specifically facilitate zinc loading into the xylem [16]. In *Arabidopsis*, expression of *AtZIP1*, *AtZIP3*, *AtZIP4*, *AtZIP5*, *AtZIP9*, *AtZIP10*, and *AtZIP12* is induced under zinc deficiency and repressed under high zinc. This regulation underscores the *ZIP* family’s role in metal ion homeostasis. Overexpression of *GmIRT1.1* in *Arabidopsis* could promote plant growth under Fe deficiency, and increase plant sensitivity to Mn and Cd excess [17]. *GmZIP1* mediates zinc translocation across the symbiotic membrane during soybean–rhizobia symbiosis [18]. Additionally, ZIP proteins contribute to abiotic stress responses. Overexpression of *OsZIP1* enhances cadmium tolerance by limiting Cd^2+^ uptake [19], and *HvIRT1* from barley transports Zn^2+^, Fe^2+^, Cd^2+^, and Mn^2+^ with broad substrate specificity [20]. In rice, *OsIRT1* enhances tolerance to saline–alkaline stress. However, the role of the *ZIP* family under alkaline stress remains poorly characterized, especially in wild soybean.

Cultivated soybean (*Glycine max*) represents a highly valuable economic crop with extensive agricultural applications [21]. However, long-term domestication imposed a genetic bottleneck on cultivated soybean [22]. Comparative genomic analyses showed that nucleotide diversity dropped by nearly half from wild soybeans to landraces [23]. Wild soybean, the progenitor of cultivated soybean, exhibits robust tolerance to multiple abiotic stresses [24]. In our previous studies, a highly adaptable alkaline-tolerant wild soybean line (G07256) was identified [25]. This line exhibits normal germination in sodic soil and maintains survival under 50 mmol/L NaHCO_3_ stress. By using transcriptome data, *ZIP* family genes were screened for differential expression under alkaline stress, identifying 17 responsive members [26]. Expression of *ZIP28* is associated with enhanced alkaline stress tolerance, while *ZIP7* is also significantly induced by alkaline stress. In this study, the *GsZIP7* gene was identified and induced by alkaline stress, zinc deficiency, and iron deficiency. Functional analyses in yeast mutants, *Arabidopsis* plants and soybean hairy roots revealed that *GsZIP7* enhanced zinc and iron deficiency tolerance and negatively regulated alkaline tolerance in plants. In total, the results suggested that *GsZIP7* negatively regulates alkaline tolerance in plants.

## 2. Results

### 2.1. Spatial and Temporal Expression Patterns of GsZIP7 in Wild Soybean

**Figure 1:**
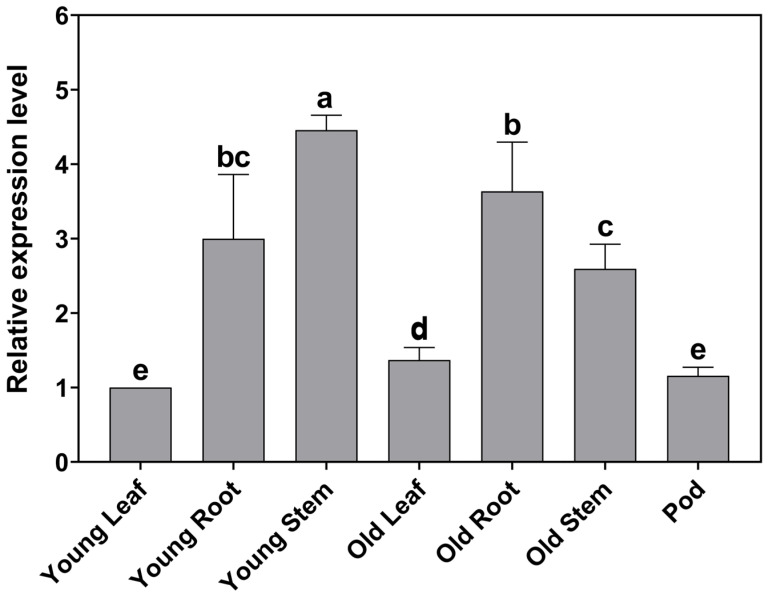
Spatial expression patterns of *GsZIP7* in different tissues of wild soybean. Expression analysis of *GsZIP7* gene in various tissues of wild soybean, including the young leaf, young root, young stem, old leaf, old root, old stem and pod. Three independent biological replicates were set for each tissue type. The relative expression level in the leaf was normalized to 1 via the 2^−∆∆Ct^ method, and the expression levels in other tissues were calculated as fold changes relative to this leaf calibrator. LSD multiple comparison test was used to test for significant differences between groups. Different letters (a–e) indicate significant differences with *p* < 0.05. All statistical calculations were completed with SPSS 21.0 software. All data visualization and graphing work was done with GraphPad Prism 8.0.1 software.

To elucidate the role of the *GsZIP7* gene in plant development, its expression patterns were examined across diverse tissues of wild soybean. The results revealed that *GsZIP7* expression was detectable in roots, stems, leaves, and pods, with varying levels (Figure 1). The highest expression was observed in young stems, while young roots, mature roots, and stems exhibited relatively high expression. In contrast, mature leaves and pods displayed significantly lower expression levels.

**Figure 2:**
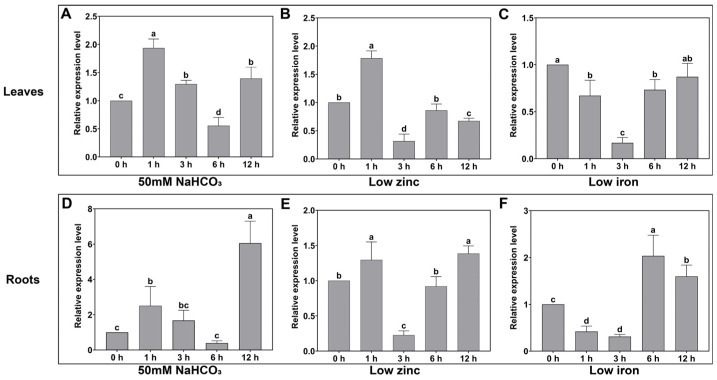
Expression levels of the *GsZIP7* gene under various stress conditions in wild soybean. (**A**,**D**) Expression analysis of *GsZIP7* gene under 50 mM NaHCO_3_ (pH 8.5) treatment. (**B**,**E**) Expression analysis of *GsZIP7* gene under low-zinc treatment. For low-zinc stress, the solution gets 0.4 mmol/L EDTA. (**C**,**F**) Expression analysis of *GsZIP7* gene under low-iron treatment. For low-iron stress, 0.02 mmol/L BPDS were added to create low-iron stress. Three independent biological replicates were set for each tissue type. The relative expression at the 0 h stress time point was normalized to 1 via the 2^−∆∆Ct^ method, and the expression levels in other stress time points were calculated as fold changes relative to this calibrator. LSD multiple comparison test was used to test for significant differences between groups. Different letters (a–d) indicate significant differences with *p* < 0.05. All statistical calculations were completed with SPSS 21.0 software. All data visualization and graphing work was done with GraphPad Prism 8.0.1 software.

To elucidate the expression characteristics of the *GsZIP7* gene in response to alkaline, low-zinc, and low-iron treatments, the expression dynamics of *GsZIP7* was detected in wild soybean roots and leaves using quantitative real-time PCR (qRT-PCR). Under alkaline stress, the expression of the *GsZIP7* peaked at 1 h, then declined with minor fluctuations in leaves (Figure 2A). In the roots, the expression of *GsZIP7* increased slightly at 1 h, reached its lowest level at 6 h, and was significantly upregulated at 12 h (Figure 2D). Under zinc deficiency treatment, the expression of *GsZIP7* rose to a maximum at 1 h and dropped to a minimum at 3 h in leaves (Figure 2B). In roots, the expression level of *GsZIP7* briefly increased at 1 h, then significantly decreased at 3 h, and peaked at 12 h (Figure 2E). The expression of *GsZIP7* was progressively suppressed until 3 h and reached its lowest level under iron deficiency stress in leaves (Figure 2C). In roots, the expression level of *GsZIP7* decreased at 1 h and 3 h, while it was significantly upregulated at 6 h and 12 h (Figure 2F). These dynamic expression patterns reveal that *GsZIP7* plays important roles in response to low iron, low zinc, and alkaline stresses.

### 2.2. Complementation Analysis in Yeast Mutant Cells

**Figure 3:**
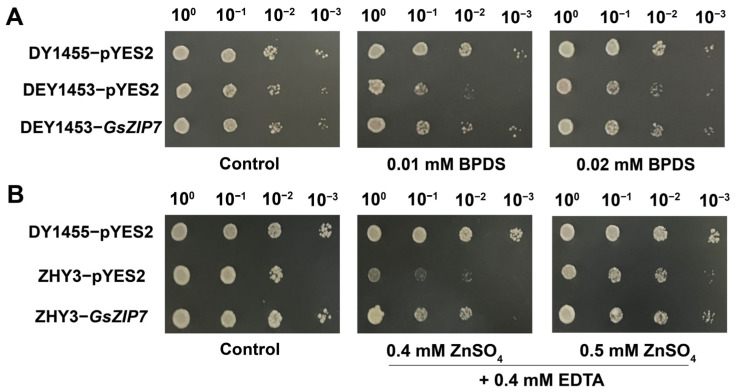
Complementation assay was performed using yeast mutants defective in zinc and iron uptake. (**A**) For iron limitation tests, SD medium was supplemented with 0.01 mM or 0.02 mM Bathophenanthroline disulfonate (BPDS). (**B**) For zinc limitation tests, SD medium was supplemented with 0.4 mM Ethylene diametetra acetic acid (EDTA) and 0.4 mM ZnSO_4_ or 0.5 mM ZnSO_4_. The zinc uptake mutant strain ZHY3 (*zrt1zrt2*), and the iron uptake mutant strain DEY1453 (*fet3fet4*). The empty pYES2 vector was transferred into WT strain DY1455 as a positive control. Plates were incubated at 30 °C for three days and then photographed to document growth.

To assess the transport function of *GsZIP7*, we performed an exogenous functional complementation assay in yeast mutants defective in zinc and iron uptake. Yeast strains harboring pYES2-*GsZIP7*, empty vector controls (ZHY3-pYES2 for zinc deficiency, DEY1453-pYES2 for iron deficiency), and wild-type DY1455-pYES2 were grown on standard SD-Ura medium or stress media supplemented with low zinc or low iron. All strains displayed comparable growth under normal conditions. The control (DEY1453-pYES2) exhibited suppressed growth, while recombinant and wild-type yeasts thrived (Figure 3A), demonstrating *GsZIP7*-mediated restoration of iron uptake in iron-limited medium. The empty vector control (ZHY3-pYES2) showed severe growth impairment, whereas *GsZIP7*-expressing recombinant and wild-type strains grew robustly, confirming functional complementation of zinc deficiency in zinc-limited medium (Figure 3B). The results showed that GsZIP7 enables efficient zinc and iron ion transport in yeast, rescuing growth defects in micronutrient-deficient mutants.

### 2.3. GsZIP7 Decreased Alkaline Tolerance in Arabidopsis

**Figure 4:**
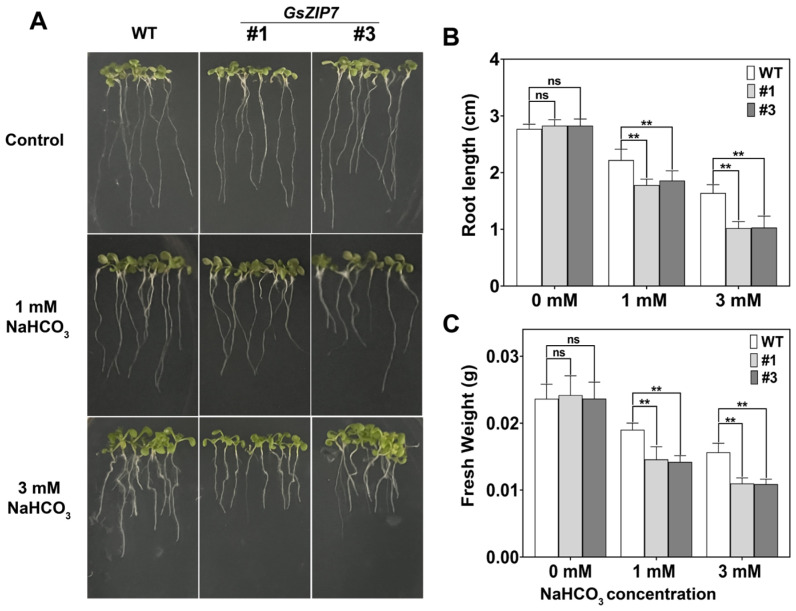
Overexpression of *GsZIP7* decreased tolerance under NaHCO_3_ stress in *Arabidopsis*. (**A**) Phenotype of *GsZIP7* transgenic lines (#1 and #3) and WT grown on 1/2-strength MS medium with the addition of 1 and 3 mM NaHCO_3_. (**B**) Root length and (**C**) fresh weight were determined under both control and alkaline stress conditions. All experimental data were processed with two-way ANOVA and Tukey’s multiple range test (*p* < 0.05). All statistical calculations were done with SPSS 21.0 software, and data visualization was completed with GraphPad Prism 8.0.1 software (ns indicates no significant difference, **: *p* < 0.01).

To elucidate the role of *GsZIP7* in responses to alkali stress, the *GsZIP7* transgenic *Arabidopsis* plants were obtained using the floral-dip method. Under standardized environmental conditions, the wild-type (WT) and *GsZIP7* transgenic plants exhibited normal growth patterns, with no statistically significant difference in root length or fresh weight. However, a significant inhibition of growth was observed in all plants compared to the control under alkali stress. *GsZIP7* transgenic plants displayed heightened sensitivity to alkali stress (Figure 4A), with root length reduced by 18% and fresh weight decreased by 24% relative to WT plants (Figure 4B,C). As the NaHCO_3_ concentration increased, the inhibitory effects on plant growth intensified. *GsZIP7* transgenic plants exhibited a more pronounced decline in root length (37.5% reduction) and fresh weight (30% reduction), compared to WT plants. These findings imply that *GsZIP7* may negatively regulate alkaline tolerance and increase stress-induced growth suppression.

### 2.4. GsZIP7 Altered Physiological Indicators and Antioxidant Enzyme Activities in Transgenic Arabidopsis Under Alkaline Stress

**Figure 5:**
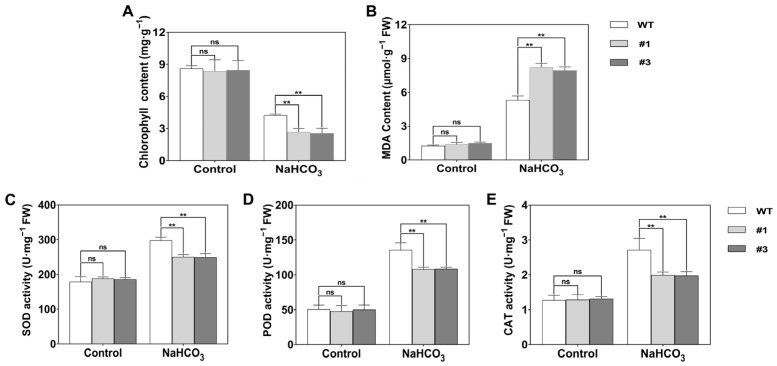
Physiological indices of *GsZIP7* under NaHCO_3_ stress treatment. (**A**) Chlorophyll, (**B**) MDA, (**C**) SOD, (**D**) POD, and (**E**) CAT enzyme activity analyses under 50 mM NaHCO_3_ stress. All experimental data were processed with two-way ANOVA and Tukey’s multiple range test (*p* < 0.05). All statistical calculations were done with SPSS 21.0 software, and data visualization was completed with GraphPad Prism 8.0.1 software (ns indicates no significant difference, **: *p* < 0.01).

To elucidate the physiological regulatory function of the *GsZIP7* gene under alkaline stress conditions, the WT and *GsZIP7* transgenic *Arabidopsis* plants were subjected to alkaline stress treatment. The results showed that alkaline stress induced a significant decrease in chlorophyll content in both WT and transgenic plants. However, chlorophyll content in the transgenic plants was 38.5% lower than that in WT, indicating a more severe disruption of photosynthesis (Figure 5A). In contrast, the malondialdehyde (MDA) level in the *GsZIP7* transgenic plants was 52% higher than that in WT, suggesting that the *GsZIP7* gene may lead to enhanced oxidative damage to the cell membrane system (Figure 5B). Alkaline stress induced the upregulation of superoxide dismutase (SOD), peroxidase (POD), and catalase (CAT) activities in both WT and *GsZIP7* transgenic *Arabidopsis* plants (Figure 5C–E). The magnitude of this increase was significantly lower in the *GsZIP7* transgenic plants compared to WT. The activity of SOD increased by 66% in WT but only 33.5% in transgenic plants. These results collectively suggest that *GsZIP7* negatively regulates the physiological regulation in response to alkaline conditions.

### 2.5. GsZIP7 Decreased Alkaline Tolerance in Soybean

**Figure 6:**
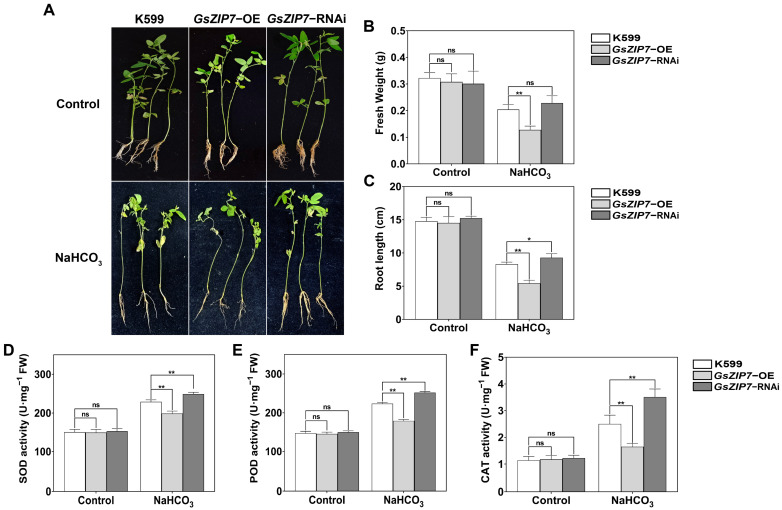
*GsZIP7* decreased tolerance under NaHCO_3_ stress in soybean hairy roots. (**A**) Phenotype of *GsZIP7* transgenic soybean hairy roots, RNAi lines and non-transgenic (K599 control) under 50 mM NaHCO_3_ treatment. (**B**) Fresh weight, (**C**) root lengths were determined under both control and alkaline stress conditions. (**D**) SOD, (**E**) POD, and (**F**) CAT enzyme activity analyses under 50 mM NaHCO_3_ stress. All experimental data were processed with two-way ANOVA and Tukey’s multiple range test (*p* < 0.05). All statistical calculations were done with SPSS 21.0 software, and data visualization was completed with GraphPad Prism 8.0.1 software (ns indicates no significant difference, *: *p* < 0.05; **: *p* < 0.01).

To further determine the functions of *GsZIP7* in response to alkaline stress, the overexpression (OE) and RNAi transgenic soybean hairy roots of *GsZIP7* were obtained. Under normal growth conditions, no significant differences were observed among the overexpression lines, RNAi lines or the K599 control. Under NaHCO_3_ stress, all lines exhibited a marked reduction in root length and fresh weight (Figure 6A). Notably, the *GsZIP7* overexpression lines displayed a more sensitive stress response, with root length and fresh weight lower than those of the control group (Figure 6B,C). In contrast, the *GsZIP7* RNAi lines demonstrated significant resistance, maintaining higher root length compared to the control group. Further, alkaline stress disrupted the normal regulation of three antioxidant enzymes (SOD, POD and CAT) in transgenic soybean hairy roots (Figure 6D–F). However, the RNAi lines showed that the enzyme activity levels were significantly higher than those in the K599 control plants. These phenotypic observations align with the growth performance of *GsZIP7*-overexpressing *Arabidopsis* under alkaline stress. This consistency indicates that *GsZIP7* negatively regulates plant tolerance in response to alkaline stress.

## 3. Discussion

The *ZIP* (zinc/iron-regulated transporter) family is essential for plant growth and development, as its members maintain cellular zinc and iron homeostasis to regulate trace nutrient uptake and utilization [27]. Studies have shown that *ZIP* family genes also participate in plant responses to abiotic stress [28]. In our previous study, the wild soybean *ZIP* family genes were identified and characterized under alkaline stress, revealing that the alkaline-responsive gene *GsZIP28* enhances plant tolerance to alkaline stress [26]. However, the role of *GsZIP7* in mediating plant alkaline stress tolerance, despite its induction under alkaline conditions, remains unelucidated. In this study, the crucial role of *GsZIP7* was detected in plant alkaline stress response, zinc deficiency and iron deficiency.

The expression analysis revealed that *GsZIP7* is constitutively expressed in various tissues in wild soybean (Figure 1). High expression in young stems and roots is indicative of a role in distributing essential micronutrients to growing and metabolically active tissues [29]. This widespread expression pattern is characteristic of many *ZIP* family transporters involved in maintaining systemic metal homeostasis [30,31]. In tea plants, several *CsZIP* genes exhibit distinct expression responses to multiple abiotic stresses and hormone treatments including NaCl, low temperature, and methyl jasmonate (MeJA) [32]. Meanwhile, *GsZIP7* is significantly upregulated in wild soybean roots under alkaline stress (Figure 2A,D), indicating its primary role in regulating abiotic stress responses in root tissues.

*ZIP* family genes can transport various metal ions, including Cd^2+^, Fe^2+^, Mn^2+^, and Zn^2+^, with substrate specificity varying among family members [33]. Zinc deficiency, iron deficiency, or deficiency of other divalent metal ions can also induce the expression of ZIP genes [34]. In this study, the results showed that the expression of *GsZIP7* was significantly induced in roots under zinc deficiency and iron deficiency stresses (Figure 2B,C,E,F). The dynamic expression of *GsZIP7* in response to zinc and iron deficiency confirms its role as a metal-responsive transporter. *ZIP3* and *ZIP5* are considered the primary transporters for root Zn uptake [35]. This aligns with the functional characterization of GsZIP7 in yeast. The growth complementation observed in both zinc uptake mutants (ZHY3-pYES2) and iron uptake mutants (DEY1453-pYES2) demonstrates that the GsZIP7 protein functions as a transporter mediating Zn^2+^ and Fe^2+^ uptake (Figure 3). *ZmZIP5* functionally complements the Zn and Fe uptake defects in the corresponding yeast mutant strains [36]. This dual-ion transport capability, observed in some *ZIP* family members, may represent a potential key node coordinating Zn^2+^ and Fe^2+^ uptake [37,38].

Under saline–alkali stress, iron tends to form insoluble oxides or hydroxides, while zinc is easily fixed by the soil, ultimately triggering iron and zinc deficiencies in plants [3,13]. *ZIP* family genes are involved in the homeostasis of various divalent metal ions, such as Mn^2+^, Ca^2+^ and Zn^2+^ [11,39,40]. Meanwhile, the *ZIP* family genes are also involved in the plant saline–alkali stress response. Previous studies revealed that the expression of *ZIP* genes is regulated in a pH-dependent manner, which modulates their metal ion transport efficiency [41]. The rice *OsIRT1* was up-regulated under Fe-deficient conditions, and enhanced tolerance to saline–alkaline stress [42,43]. In this study, *GsZIP7* was also induced under alkaline stress and mediated the transport of zinc and iron ions in plants. However, after alkali stress, the *GsZIP7* transgenic *Arabidopsis* lines exhibited reduced tolerance compared to the WT, as evidenced by shorter root lengths and decreased fresh biomass (Figure 4). *GsZIP7* transgenic soybean hairy root lines also exhibited more severe growth inhibition under NaHCO_3_ stress compared to WT and RNAi lines, while the RNAi lines showed enhanced tolerance (Figure 6). However, the current research results are inconsistent with our previous studies on *ZIP* family genes, which demonstrated that *GsZIP28* enhances alkaline stress tolerance in soybean [26]. In wheat, *TaZIP13-B*, *TaZIP14-B*, and *TaIRT2-A* function as functional Zn^2+^/Fe^2+^ transporters, while salt stress suppresses their Zn/Fe transport capacity [44]. Members of the *ZIP* family in foxtail millet may be involved in the intra-cellular redistribution of metal ions such as Zn^2+^ and Fe^2+^ under salt stress [45]. In this study, under alkaline stress, it is hypothesized that the altered transport activity of *GsZIP7* for Zn^2+^/Fe^2+^ may inadvertently distort these critical ionic signals or create ion imbalances [46].

Under salt stress, exogenous Zn or ZnO nanoparticles can alleviate salt-induced damage, mainly by reducing the accumulation of reactive oxygen species (ROS) and MDA, and enhancing the activities of antioxidant enzymes [47,48,49]. In kidney beans, ZnO nanoparticles have been demonstrated to mitigate salt stress damage by improving chlorophyll fluorescence parameters [47]. In our previous study, *GsZIP28*-overexpressing lines exhibited enhanced tolerance under alkaline stress, which was mainly manifested as reduced superoxide anion accumulation and strengthened antioxidant defense capacity [26]. Overall, salt or alkali stress triggers excessive ROS accumulation and reduces the bioavailability of Zn and Fe. Subsequently, *ZIP/IRT* family genes mediate the absorption and redistribution of metal ions such as Zn^2+^ and Fe^2+^. By maintaining Zn/Fe homeostasis, these genes preserve chlorophyll synthesis, photosynthetic efficiency, antioxidant enzyme activities and cellular redox balance, ultimately enhancing plant adaptability to salt or alkali stress. However, in this study, the results showed that transgenic plants displayed lower chlorophyll content and higher MDA levels than WT plants under alkaline stress (Figure 5A,B), indicating amplified damage to the photosynthetic and cellular membranes from lipid peroxidation [50,51]. As expected, SOD, POD and CAT showed a smaller activity increase in transgenic plants under stress, compared to WT (Figure 5C–E). The RNAi lines of *GsZIP7* showed that the enzyme activity levels were significantly higher than those in control plants in hairy roots (Figure 6D–F). It is hypothesized that *GsZIP7* may disrupt cellular Zn and Fe homeostasis, resulting in chlorophyll degradation, reduced antioxidant enzyme activities, and ultimately increased plant sensitivity to alkaline stress.

## 4. Conclusions

Taken together, the *GsZIP7* gene in wild soybean encodes a functional zinc and iron transporter. While this transporter is essential for micronutrient acquisition, *GsZIP7* overexpression confers sensitivity to alkaline stress by likely interfering with the activation of antioxidant enzymes. However, future investigations on the responses of *GsZIP7* transgenic plants to zinc- and iron-deficiency stresses are essential. Also, it will be crucial to investigate the proteins that interact with GsZIP7, which may reveal its integration point into stress signaling networks. In addition, *ZIP7* loss-of-function lines should be generated in cultivated soybean using CRISPR/Cas9, and the performance of these lines should be assessed under alkaline field conditions. This approach can provide a direct breeding strategy for improving alkaline soil tolerance.

## 5. Materials and Methods

### 5.1. Expression Analysis of GsZIP7

The wild soybean seeds G07256 were treated with concentrated sulfuric acid for 12–15 min. The treatment was done under constant shaking to break the hard seed coat. After the acid treatment, the seeds were rinsed four times with sterile distilled water and then moved onto moist filter paper for two days. The young seedlings were moved to a hydroponic culture device with Hoagland nutrient solution (KNO_3_ 2.09 mol/L, NH_4_H_2_PO_4_ 0.2 mol/L, Ca(NO_3_)_2_·4H_2_O 0.8 mol/L, MgSO_4_·7H_2_O 0.4 mol/L, EDTA-Fe 61.6 mmol/L, H_3_BO_3_ 46.26 mmol/L, MnCl_2_·4H_2_O 9.1 mmol/L, ZnSO_4_·7H_2_O 0.765 mmol/L, CuSO_4_·5H_2_O 0.316 mmol/L, (NH_4_)_6_Mo_7_O_24_·4H_2_O 0.03 mmol/L), adjusted to pH 5.8 [52]. The nutrient solution was changed once every 72 h. All plants were grown in a controlled growth chamber. The temperature stayed between 22 °C and 28 °C. The growth environment was maintained at 60–65% relative humidity and 320 μmol photons m^−2^ s^−1^. The daily light cycle was set to 16 h of light followed by 8 h of dark. To analyze expression levels in different tissues and organs, the young leaf, root and stem were collected from 21-day-old seedlings. The old leaves, roots, stems and pods were collected from three-month-old plants.

For alkali, low-zinc, and low-iron stress treatments, the 21-day-old seedlings were placed in Hoagland nutrient solution for each treatment. Different additives were added to the Hoagland solution for each treatment. A total of 50 mM NaHCO_3_ (pH 8.5) was added for alkali stress. For low-zinc stress, 0.4 mmol/L EDTA was added to the nutrient solution. A total of 0.02 mmol/L BPDS was added to create low-iron stress. The root and leaf tissue samples were collected at five set time points: 0, 1, 3, 6, and 12 h. Total RNA was extracted from every tissue sample using a commercial RNA extraction kit (Omega Bio-Tek, Beijing, China). The reverse transcription kit (Thermo Fisher, Waltham, MA, USA) was used to synthesize cDNA. *GsGAPDH* was chosen as the reference gene for this experiment (Table S1). Three independent biological replicates were set for each tissue type. The 2^−∆∆Ct^ method was used to calculate relative gene expression. The LSD multiple comparison test was used to test for significant differences between groups. All statistical calculations were completed with SPSS 21.0 software. All data visualization and graphing work was done with GraphPad Prism 8.0.1 software.

### 5.2. Analysis of Zinc and Iron Ion Transport Capacity in GsZIP7 Transgenic Yeast

The full-length coding sequence of *GsZIP7* was inserted into the yeast expression vector pYES2. This experiment used three different yeast strains. The strains were the WT strain DY1455, the zinc uptake mutant strain ZHY3 (*zrt1zrt2*), and the iron uptake mutant strain DEY1453 (*fet3fet4*). The empty pYES2 vector was transformed into the WT strain DY1455 as a positive control. The empty pYES2 vector and the pYES2-*GsZIP7* recombinant vector were separately transformed into the mutant strains ZHY3 and DEY1453. To test metal transport function, the yeast strains were grown on low-zinc or low-iron media. For zinc limitation tests, SD medium was supplemented with 0.4 mM EDTA and 0.4 mM ZnSO_4_ or 0.5 mM ZnSO_4_ [53]. For iron limitation tests, SD medium was supplemented with 0.01 mM or 0.02 mM BPDS [54]. Plates were incubated at 30 °C for three days and then photographed to document growth.

### 5.3. Phenotypic Analysis of GsZIP7 in Arabidopsis Under Alkali Stress

The gene-specific primers were used to amplify the full-length coding sequence of *GsZIP7* (Table S1). The amplified GsZIP7 full-length coding sequence was inserted into the pCAMBIA230035S vector. The recombinant vector was transferred into *Agrobacterium tumefaciens* strain GV3101. Then the floral dip method was used to generate transgenic *Arabidopsis* plants (Figure S1) [55]. All *Arabidopsis* plants were grown in a controlled greenhouse. The growth environment followed 20–23 °C, 60–65% relative humidity, and 100 μmol photons m^−2^ s^−1^, with 16 h light/8 h dark daily photoperiod. The transgenic lines were screened on half-strength MS solid medium with 25 mg L^−1^ kanamycin. Two homozygous T_3_ transgenic lines, labeled #1 and #3, were randomly picked for downstream phenotypic analysis. For alkali stress treatment, seeds of transgenic lines and WT *Arabidopsis* were sown on different 1/2-strength MS media supplemented with three distinct NaHCO_3_ concentrations: 0, 1, and 3 mM. After two weeks of growth, the total root length and whole plant fresh weight were measured. All experiments were carried out with at least three independent biological replicates, with at least six seedlings included per replicate.

### 5.4. Phenotypic Analysis of GsZIP7 in Soybean Hairy Roots Under Alkali Stress

The *Glycine max* cultivar Dongnong 50 was used as plant material. All soybean seedlings were grown in a plant light incubator (Xinyi Company, Shanghai, China). The growth conditions followed a 16 h light/8 h dark daily photoperiod. Five-day-old young soybean seedlings were selected for inoculation. The 300 bp optimal silencing fragment targeting the *GsZIP7* gene was designed using the online SGN VIGS Tool (https://vigs.solgenomics.net/) [56]. The target fragment was ligated into the empty pTRV2 vector to complete the RNAi vector construction. The *Agrobacterium rhizogenes* strain K599 carried the pCAMBIA230035S:*GsZIP7* or pTRV2-RNAi-*GsZIP7* recombinant construct. The inoculated seedlings grew new hairy roots from the infection site until the hairy roots reached 3–4 cm in length (Figure S1). Then, the whole plants were transferred to pots filled with sterile vermiculite. Plants were treated with 50 mM NaHCO_3_ solution to induce alkaline stress. After two weeks, the root length and total root fresh weight of the soybean hairy roots were measured. All experiments were carried out with at least 15 seedlings per experiment.

### 5.5. Analysis of Physiological Indicators of GsZIP7 in Arabidopsis and Soybean Hairy Roots Under Alkali Stress

For physiological index analysis, the two homozygous *GsZIP7* transgenic lines (#1 and #3) and WT *Arabidopsis* were used as materials. All *Arabidopsis* plants were grown in a mixture of soil and vermiculite at a 1:1 ratio. The growth environment followed a 16 h light/8 h dark daily photoperiod. After 50 mM NaHCO_3_ alkaline stress treatment, the leaf samples were collected from both control and stress groups. The hairy roots were treated with 50 mM NaHCO_3_ for two weeks and collected from both control and stress groups. Different standard methods were used to test each physiological indicator. Chlorophyll content was measured with a spectrophotometric method [57]. MDA content was measured with the thiobarbituric acid colorimetric method [58]. POD activity was tested with the guaiacol method [59]. SOD activity was measured with the nitroblue tetrazolium method [60]. CAT activity was quantified with the ultraviolet absorption method [61]. All experimental data were processed with two-way ANOVA and Tukey’s multiple range test (*p* < 0.05). All statistical calculations were done with SPSS 21.0 software, and data visualization was completed with GraphPad Prism 8.0.1 software.

## Data Availability

The datasets generated and analyzed during the current study are available from the corresponding author on reasonable request.

## Supplementary Materials

The following supporting information can be downloaded at: https://www.mdpi.com/article/10.3390/plants15142152/s1, Table S1: Gene-specific primers used in this study. Figure S1. Identification of *GsZIP7* transcript levels in transgenic *Arabidopsis* and soybean hairy roots. (**A**) RT-PCR analysis of *GsZIP7* expression in transgenic *Arabidopsis*. (**B**) qRT-PCR analysis of *GsZIP7* expression in soybean hairy roots. The relative expression level in the K599 (control) was normalized to 1 via the 2^−ΔΔCt^ method, and the expression levels in other transgenic lines were calculated as fold changes relative to this calibrator. Significant differences were determined using Student’s *t*-test. Statistical analyses were performed with SPSS 21.0. Asterisks in the figure denote statistical significance between groups (**: *p* < 0.01).

## Abbreviations

The following abbreviations are used in this manuscript:

ZIPs	Zinc/iron-regulated transporter proteins
TMs	Transmembrane domains
BPDS	Bathophenanthroline disulfonate
EDTA	Ethylene diametetra acetic acid
WT	Wild-type
OE	Overexpression
MeJA	Methyl jasmonate
MDA	Malondialdehyde
SOD	Superoxide dismutase
CAT	Catalase
POD	Peroxidase
ROS	Reactive oxygen species
qRT-PCR	Quantitative real-time PCR
