# Supplementary material for: GsZIP7, a Zinc/Iron-Regulated Transporter Protein from Wild Soybean, Confers Enhanced Sensitivity to Alkaline Stress
Source: Plants (Basel). 2026 Jul 13;15(14):2152. doi: 10.3390/plants15142152 (PMC13416046; doi:10.3390/plants15142152)
Supplement: Supplementary file 1 [file plants-15-02152-s001.zip › Figure S1.pdf]

Supplementary Figure S1. Identification of *GsZIP7* transcript levels in transgenic *Arabidopsis* and soybean hairy roots.

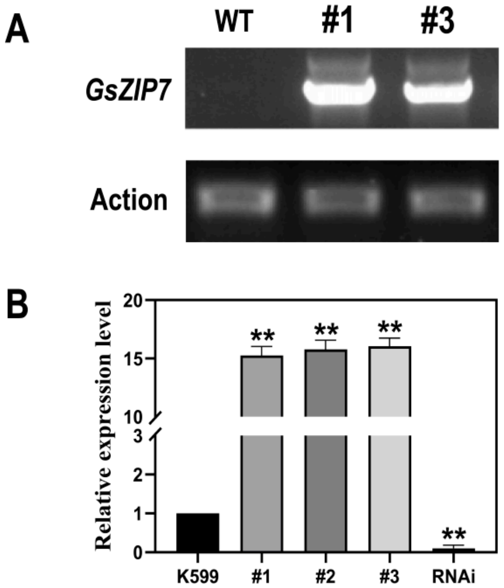

Figure S1. Identification of *GsZIP7* transcript levels in transgenic *Arabidopsis* and soybean hairy roots. **(A)** RT-PCR analysis of *GsZIP7* expression in transgenic *Arabidopsis*. **(B)** qRT-PCR analysis of *GsZIP7* expression in soybean hairy roots. The relative expression level in the K599 (control) was normalized to 1 via the $2^{-\Delta\Delta Ct}$ method, and the expression levels in other transgenic lines were calculated as fold changes relative to this calibrator. Significant differences were determined using Student's *t*-test. Statistical analyses were performed with SPSS 21.0. Asterisks in the figure denote statistical significance between groups (**: *p* <0.01).
