# Supplementary material for: GsZIP7, a Zinc/Iron-Regulated Transporter Protein from Wild Soybean, Confers Enhanced Sensitivity to Alkaline Stress
Source: Plants (Basel). 2026 Jul 13;15(14):2152. doi: 10.3390/plants15142152 (PMC13416046; doi:10.3390/plants15142152)
Supplement: Supplementary file 1 [file plants-15-02152-s001.zip › Table S1.pdf]

Supplementary Table S1 Gene-specific primers used in this study.

Gene ID	Primer sequence (5′-3′)
GADPH	Forward: GACTGGTATGGCATTCCGTGT
	Reverse: GCCCTCTGATTCCTCCTTGA
GsZIP7(For qRT-PCR)	Forward: GTCTACGGTAATTATGGCGACC
	Reverse: TTTTCTGCAACTTTGGGCTC
GsZIP7 (For gene clone)	Forward: TTCACTCACTTAATTGTGCAATCC
	Reverse: TAAAGTAAAACACCAAAATTAGGCC
GsZIP7 (For RNAi)	Forward: GCGAATTCAGCAAGAGAATCCCAACAC
	Reverse: GCTCTAGTTTCATCTCCCATTTCCTCATC
Actin	Forward: TTACCCGATGGGCAAGTC
	Reverse: GCTCATACGGTCAGCGATAC
